# Interventional embolization therapy of puerile congenital deep femoral arteriovenous fistula

**DOI:** 10.3892/etm.2012.832

**Published:** 2012-11-26

**Authors:** JING ZHANG, XIAO-YUN TAN, SHAO-YI ZHOU, KUN-SHAN CHEN, HAI-BO LI, CHUANQIANG-NIU, YI-ZHOU JIANG, QUE-QING LIN

**Affiliations:** Department of Interventional Therapy, Guangzhou Women and Children's Medical Center, Guangzhou 510120, P.R. China

**Keywords:** congenital arteriovenous fistula, embolization, spring steel ring, absolute ethyl alcohol, intervention

## Abstract

This study aimed to investigate the treatment efficiency of interventional embolization therapy in puerile congenital deep femoral arteriovenous fistula. A retrospective analysis was conducted for 9 cases of congenital deep femoral arteriovenous fistulae treated in our department in the past 5 years. B-ultrasound examination indicated that all puerile patients suffered from deep femoral arteriovenous fistulae, which was confirmed by angiography examination. For all patients, endovascular interventional embolization therapy was conducted and angiography re-examination was implemented after 4 weeks. If there were residual orificium fistulae, the interventional embolization therapy was conducted again. In the 6 month to 2 year follow-up period, improvement of clinical symptoms was observed. Following interventional embolization, 9 cases of deep femoral arteriovenous fistulae were completely occluded and the clinical symptoms were improved. No relapses occurred. In addition, after three embolization treatments, the disease condition of one case was controlled well and the disease condition did not progress. Interventional embolization therapy has a number of advantages, including simple surgery and reliable treatment efficacy. Therefore, it is worthy of promotion and application in the clinic.

## Introduction

Cases of arteriovenous fistula include congenital and acquired arteriovenous fistulae. Acquired arteriovenous fistula is common in traumas and iatrogenic surgery, including acupuncture, moxibustion and femoral artery puncture (1–5). Puerile congenital arteriovenous fistula is rare in the clinic and it may occur at any site of body, including coronary arteriovenous, dural arteriovenous, spinal peripheral arteriovenous and pulmonary arteriovenous fistulae (6,7). Additionally, reports on puerile congenital deep femoral arteriovenous fistula are rare and its treatment methods mainly depend on surgery (8). With the development of intervention technology in China in the past 20 years, interventional embolization is increasingly applied in the treatment of arteriovenous fistula (9–11). However, there are still no reports on interventional embolization as a treatment of puerile congenital deep femoral arteriovenous fistula. In this study, we used interventional embolization to treat 9 cases of puerile congenital deep femoral arteriovenous fistulae to obtain improved treatment efficiency.

## Materials and methods

### General data

This study was conducted in accordance with the declaration of Helsinki. This study was conducted with approval from the Ethics Committee of the Guangzhou Women and Children's Medical Center, and written informed consent was obtained from all participants. A total of 9 patients, 6 male and 3 female, were included in this study. Patient ages ranged from 4 to 15 years and their mean age was 10.7 years. Body weight ranged from 16 to 49 kg and the mean body weight was 24.7 kg. Additionally, no patients had explicit trauma history. In 3 cases, the lesions were located in the left thigh and in 6 cases the lesions were located in the right thigh. For all 9 patients, fistula skin temperature had increased and the skin temperature at the affected side was higher (maximum increase, 1.5°C) compared to that at the the same site on the healthy side. Seven patients presented local skin hyperhidrosis; 2 patients presented red spots on the skin surface; 5 patients presented palpable vibratory sense of lesions; 3 patients presented apparent pulsatory sense; 3 patients suffered from superficial varicose veins on the root of the thigh (caused by standing); 3 patients suffered from local thickening of the thigh, including 1 patients who suffered from local thickening of the thigh accompanied with mild atrophy of the calf muscle; 2 patients presented affected limb elongation, by a maximum of 1.5 cm; 5 patients often felt limb numbness, swelling or pain and in 2 patients these symptoms were occasionally accompanied by pelvic cavity and lower back pains. In addition, all 9 patients had no clear palpitation after activity and no skin ulcers or gangrene occurred in the affected limb. Color Doppler B-ultrasound examination was conducted for all 9 patients to ensure correct diagnosis of arteriovenous fistula. Selective angiography via femoral artery approach was also conducted. The angiography results revealed that the femoral vein developed in advance of the deep femoral artery. The orificium fistulae were located at the proximal end of the deep femoral artery or the distal end of its branch. According to Vollmar typing, 6 cases were type I, 2 cases were type II and 1 case was type III.

### Treatment methods

The femoral artery at the healthy side was punctured with a 4F pediatric puncture sheath kit (Terumo Company, Tokyo, Japan) by the Seldinger technique. After guide wires were exchanged, a 4F catheter (Terumo Company) was placed into the common iliac artery at the affected limb and radiography was carried out. After determining the location of the fistula, a 2.7F microcatheter (Terumo Company) was inserted into the orificium fistula and its location was repeatedly confirmed by radiography. Subsequently, embolism materials, including spring steel rings or absolute ethyl alcohol, were injected into the vessel. Occlusion of the fistulae branches was performed by locally puncturing lesions. After 15 min, the femoral angiography was conducted again. If there were still residual orificium fistulae, fistulae branches were continuously occluded by the same method until orificium fistulae were completely closed. One month after surgery, a re-examination was conducted. If necessary, the interventional embolization therapy was conducted again.

## Results

### Angiography

**Figure 1.**
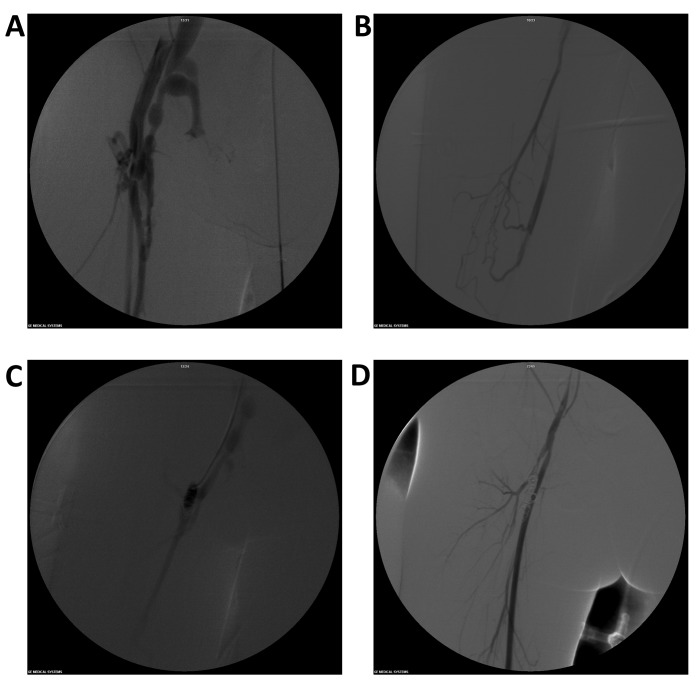
Congenital right deep femoral arteriovenous fistula (female, 7 years). (A) Deep femoral arteriovenous fistula, arteriectasia at the orificium fistula segment and venous tortuosity at the orificium fistula end. A number of branches of the deep femoral artery had a poor display due to ischemia; however, the lateral femoral circumflex artery lower branch dilated. (B) Deep femoral artery, lateral femoral circumflex artery and lower branch fistulae. Absolute ethyl alcohol was injected for treatment. (C) After orificium fistula location was determined, spring steel rings were released. (D) After one year, re-examination was conducted. Orificium fistulae were completely occluded and the developing deep femoral artery branch was clear.

Angiography clearly showed the state of the deep femoral artery and its branches, as well as the orificium fistulae (Fig. 1A). Among the 9 cases of congenital arteriovenous fistula (CAVF) in this study, 4 cases presented a single orificium fistula and 5 cases presented a number of orificium fistulae. The main orificium fistulae were located in the trunk of the deep femoral artery, 1–3 cm away from the deep femoral artery opening. The branches of the orificium fistulae of the deep femoral artery were finer (Fig. 1B). The proximal arteries and veins of all 9 cases of orificium fistulae presented varying extents of dilation and distortion. Among them, the proximal deep femoral arteries in 6 cases of orificium fistulae presented tumor-like dilation accompanied with deep femoral artery branch dilation. The distal arteries and veins in 4 cases of orificium fistulae were almost normal. The distal veins in 2 cases of orificium fistulae presented slow blood backflow and reverse flow and the backflow in the vein in 1 case flowed towards the pelvic cavity.

### Operative techniques and fistula occlusion

A total of 11 interventional embolization treatments were conducted for 9 patients and a total of 47 spring steel rings were released. Technical surgery success rate was 100% (Fig. 1C and D) and no ectopic embolism of spring steel ring occurred. The intraoperative immediate fistula occlusion rate reached 100%. One month after surgery, angiography re-examination was conducted again and the fistula occlusion rate was 88.9% (8/9 cases). Among them, numerous tiny orificium fistulae were visible in the branches of the deep femoral artery in 1 case. A total of three successive interventional embolizations were conducted for the patient.

### Improvement of clinical symptoms and follow-up

Compared with before treatment, skin temperature at the affected side in 9 patients was reduced. Six cases were almost normal; pulsatory or vibratory sense in 7 cases disappeared; 1 case presented palpable mild fremitus and the orificium fistulae location was unclear; the affected limb in 1 case was occasionally accompanied with pain, and superficial varicosis on the thigh of 1 case was still visible; however, it was milder than before surgery. During hospitalization, no surgical complications, including cutaneous necrosis occurred. Within the postoperative period of 6 months to 2 years, 8 cases reached the clinical criteria for being cured. In addition, although 1 case still experienced CAVF symptoms, the disease condition did not progress more clearly compared to before surgery.

## Discussion

CAVF lesions are occasionally present at birth; however, after inquiring about disease history, we noted that patients had no clinical symptoms. Due to this, CAVF does not attract attention and therefore has a longer latency. Usually there are clear manifestations in the school age or adolescent period, which cause concern. Of the 9 patients in this study, symptoms in 3 cases appeared in the school age period and symptoms in 6 cases appeared during the adolescent period, which is possibly associated with endocrine hormone stimulation, over-activity or trauma (although puerile patients had no clear trauma history) in this stage (12).

Although a number of classification methods are available for CAVF, the Vollmar classification method is the most common in the clinic. In 1976, Vollmar divided CAVF into three types according to the morphology: type I (trunk-like arteriovenous fistula), traffic branches on the horizontal direction among peripheral arteriovenous trunks; type II (tumor-like arteriovenous fistula), a number of fine traffic branches on the horizontal direction among peripheral arteriovenous involving trunks and local soft tissues and skeletons; and type III (mixed mode), trunk- and tumor-like multiple arteriovenous traffic branches. The Vollmar classification method represents the occurrence and development process of CAVF and its complexity. In this study, 6 cases were type I, with angiography clearly showing the orificium fistula location and the progressive dilation and tortuosity of arteries and veins at the proximal orificium fistula. The arteries and veins at the distal orificium fistula were almost normal and the majority presented palpable fremitus. Auscultation identified a ‘purring thrill’ sound and a number of cases presented palpable pulsation. Two cases were type II and angiography showed multiple branches of the fistula sinus, increased arterial and venous collateral cycle and an evident thickening of local soft tissue. Additionally, the vibratory sense was wide; however, the location was unclear. One case was type III and angiography showed tumor-like dilation at the proximal orificium fistulae of the deep femoral artery accompanied with deep femoral artery branch dilation. At the distal end, several microfistulae were visible and back-flow veins were tortuous. Additionally, partial venous valve function insufficiency and distal venous blood stasis were visible and reverse blood flow was occasionally visible. In the clinic, extended and thickened limbs with superficial varicosis at the affected side were evident. Pain symptoms in the lower extremities and pelvic cavity were also more apparent.

Although CAVF is a benign lesion, it has the biological behaviors of a malignant tumor. Therefore, the lesion continuously develops, spreads and often involves adjacent tissues and organs, inducing severe complications, including affected limb swelling, thickening, pain, hyperhidrosis, pigmentation, festering, necrosis and congestive heart failure. The cases in this study showed no severe complications, including limb necrosis and congestive heart failure, which may be associated with earlier identification of the lesion and timely clinical treatment. A number of studies have reported that after adulthood, CAVF presents various severe complications, including the steal syndrome, intractable ulcers and progressive heart failure (6,7). As CAVF has no self-healing tendency, diagnosis and treatment must be conducted as early as possible. However, clinical treatment is difficult. From the perspective of hemodynamics, CAVF is an abnormal communication between the high-pressure and high-resistance arterial system and the low-pressure, low-resistance and high-capacity venous system. Surgical ligation is difficult but it completely clears lesions. Usually, the ligation end is too far away from the orificium fistula or numerous microfistulae are present. Surgery only ligates the main fistula branches, while post-operative fine fistula branches may either gradually dilate or the collateral artery may enter the fistula cycle, inducing lesion recurrence. Particularly for complex CAVF, treatment often begins with surgery or embolism and ends with amputation (13).

**Figure 2.**
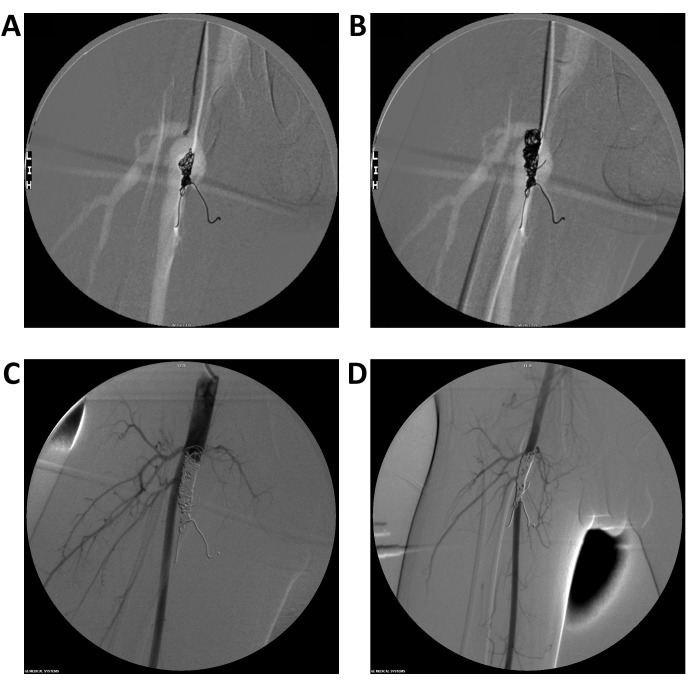
Congenital right deep femoral arteriovenous fistula (male, 12 years). (A) Right deep femoral arteriovenous fistula and tumor-like dilation complicated with deep femoral artery branch dilation at the orificium fistula end. Backflow veins developed in advance and the fistula sinus blood flow rate was rapid. (B) The catheter was inserted into the orificium fistula and spring steel rings were anchored onto any branch of the orificium fistula and gradually filled. (C) Spring steel rings were continuously filled and an appropriate amount of absolute ethyl alcohol was injected. (D) Re-examination was performed after 6 months. Orificium fistulae were completely occluded and the superficial femoral artery development was good. No limbs of the patients presented ischemic necrosis.

CAVF is the result of long-term development of vascular abnormalities. In contrast to traumatic or iatrogenic arteriovenous fistula, the majority of CAVF, other than the main arteriovenous fistula, usually present varying extents of minute orificium fistulae at the main trunk branches. Therefore, a high pressure injector is used and angiography examination clearly shows the effects. In this study, 5 cases presented varying amounts of microfistulae, while surgical ligation readily caused recurrence. We used the spring steel rings plus absolute ethyl alcohol embolization treatment method (14,15) and obtained improved efficiency. The one-time interventional surgery fistula occlusion rate in 8 cases reached 100%. Following interventional surgery in 1 case, belonging to type III, orificium fistulae formed again. After three interventional embolization treatments, the disease conditions were better controlled. We consider that the following should be noted for surgery: spring steel rings create a permanent embolism. Before releasing spring steel rings, it is necessary to ensure that no other fistula exists at the distal end of this branched artery by repeating angiography. Otherwise, it is possible to aggravate the disease condition and it becomes impossible to conduct subsequent treatments as there is no vascular channel. Additionally, before spring steel rings are released, it is necessary to ensure the opening end of the catheter is at the orificium fistula or in the sinus fistula, which may be confirmed by radiography, i.e., conducting backflow vein development of the orificium fistula following injection of a contrast agent and then increasing pressure again. As a result, the deep femoral artery back-flow developing at the proximal orificium fistula is visible. To ensure the embolism level reaches the orificium fistula grade, fistula cycling in the collateral branch must be avoided, as this causes lesion recurrence. For the small branch or residual orificium fistulae following the release of the spring steel rings, it is possible to occlude residual lesions by injecting absolute ethyl alcohol via an endovascular injection or local puncture of the lesions. A larger orificium fistula usually requires more spring steel rings. If the blood flow of the orificium fistula is larger, it is possible to firstly fix spring steel rings onto any branch near the orificium fistula by the anchoring method (Fig. 2A and B). On this base, spring steel rings are gradually and densely filled and the high-flow rate fistula tract becomes a low-flow rate fistula tract. Afterwards, residual mesh-like, microfistulae are occluded by injecting absolute ethyl alcohol (Fig. 2C and D). However, absolute ethyl alcohol injection may induce acute hemolysis, cutaneous necrosis and pulmonary hypertension; therefore it may be necessary to conduct pulmonary artery pressure monitoring. Additionally, it is recommended that surgery is conducted by an experienced senior interventional physician (16–18).

To conclude, puerile congenital deep femoral arteriovenous fistula sustainably progresses, so it must be diagnosed and treated as early as possible. Interventional embolization has a number of advantages, including small trauma, little complication and clear efficiency and is increasingly applied in the clinic. Therefore, it may become the preferred therapeutic regimen for puerile congenital deep femoral arteriovenous fistula.
